# MolFeSCue: enhancing molecular property prediction in data-limited and imbalanced contexts using few-shot and contrastive learning

**DOI:** 10.1093/bioinformatics/btae118

**Published:** 2024-02-29

**Authors:** Ruochi Zhang, Chao Wu, Qian Yang, Chang Liu, Yan Wang, Kewei Li, Lan Huang, Fengfeng Zhou

**Affiliations:** Key Laboratory of Symbolic Computation and Knowledge Engineering of Ministry of Education, Jilin University, Changchun, Jilin 130012, China; School of Artificial Intelligence, Jilin University, Changchun 130012, China; Key Laboratory of Symbolic Computation and Knowledge Engineering of Ministry of Education, Jilin University, Changchun, Jilin 130012, China; College of Computer Science and Technology, Jilin University, Changchun, Jilin 130012, China; Key Laboratory of Symbolic Computation and Knowledge Engineering of Ministry of Education, Jilin University, Changchun, Jilin 130012, China; College of Computer Science and Technology, Jilin University, Changchun, Jilin 130012, China; Beijing Life Science Academy, Beijing 102209, China; Key Laboratory of Symbolic Computation and Knowledge Engineering of Ministry of Education, Jilin University, Changchun, Jilin 130012, China; College of Computer Science and Technology, Jilin University, Changchun, Jilin 130012, China; Key Laboratory of Symbolic Computation and Knowledge Engineering of Ministry of Education, Jilin University, Changchun, Jilin 130012, China; College of Computer Science and Technology, Jilin University, Changchun, Jilin 130012, China; Key Laboratory of Symbolic Computation and Knowledge Engineering of Ministry of Education, Jilin University, Changchun, Jilin 130012, China; College of Computer Science and Technology, Jilin University, Changchun, Jilin 130012, China; Key Laboratory of Symbolic Computation and Knowledge Engineering of Ministry of Education, Jilin University, Changchun, Jilin 130012, China; College of Computer Science and Technology, Jilin University, Changchun, Jilin 130012, China; School of Biology and Engineering, Guizhou Medical University, Guiyang, Guizhou 550025, China

## Abstract

**Motivation:**

Predicting molecular properties is a pivotal task in various scientific domains, including drug discovery, material science, and computational chemistry. This problem is often hindered by the lack of annotated data and imbalanced class distributions, which pose significant challenges in developing accurate and robust predictive models.

**Results:**

This study tackles these issues by employing pretrained molecular models within a few-shot learning framework. A novel dynamic contrastive loss function is utilized to further improve model performance in the situation of class imbalance. The proposed MolFeSCue framework not only facilitates rapid generalization from minimal samples, but also employs a contrastive loss function to extract meaningful molecular representations from imbalanced datasets. Extensive evaluations and comparisons of MolFeSCue and state-of-the-art algorithms have been conducted on multiple benchmark datasets, and the experimental data demonstrate our algorithm’s effectiveness in molecular representations and its broad applicability across various pretrained models. Our findings underscore MolFeSCues potential to accelerate advancements in drug discovery.

**Availability and implementation:**

We have made all the source code utilized in this study publicly accessible via GitHub at http://www.healthinformaticslab.org/supp/ or https://github.com/zhangruochi/MolFeSCue. The code (MolFeSCue-v1-00) is also available as the supplementary file of this paper.

## 1 Introduction

Accurate detection of molecular properties is a critical task for drug discovery and material science (Butler *et al.* 2018). Computational prediction of these properties enables researchers and engineers to efficiently design novel therapeutics and materials, optimize existing molecules, and understand complex molecular interactions (Goh *et al.* 2017). Despite the vast progress in machine learning and deep learning techniques, predicting molecular properties remains a challenging endeavor (Cheng *et al.* 2013, Huang *et al.* 2021) with the difficulties of data scarcity and class imbalance (Dearden 2007, Aleksić *et al.* 2022).

Accurate prediction models often require large quantities of annotated data to achieve satisfactory performance (Chen and Lin 2014). However, obtaining a large labeled dataset for molecular properties is infeasible in many practical cases due to the high cost of generating experimental validation data or the inherent rarity of certain properties (Paul *et al.* 2021). Few-shot learning is a promising paradigm to overcome the data scarcity problem by training models to generalize from a limited number of samples (Wang *et al.* 2020). This approach leverages prior knowledge to adapt to new tasks with a few samples, thereby enabling robust predictions in situations with insufficient training data (Sun *et al.* 2019).

Class imbalance can be tackled by contrastive learning (Liu, 2023b), which is an unsupervised technique learning to encode useful representations by contrasting similar and dissimilar samples (Jaiswal *et al.* 2020). Contrastive learning has already been employed to extract meaningful features from molecular structures and capture intrinsic relationships among molecules that share specific properties (Pinheiro *et al.* 2022, Wang *et al.* 2022). The subtle differences between molecules with different properties may be further amplified by contrastive learning, which is crucial for addressing the issue of highly imbalanced class distribution.

This study proposes a novel few-shot contrastive learning framework MolFeSCue for predicting molecular properties in the context of both data scarcity and class imbalance. We utilize three pretrained models as molecular representations in our MolFeSCue framework, and demonstrate that MolFeSCue can effectively learn molecular representations across diverse situations. We evaluate the performance of MolFeSCue on various benchmark datasets, and MolFeSCue outperforms the current state-of-the-art approaches. The proposed MolFeSCue framework offers a robust solution for predicting molecular properties in challenging situations of data scarcity and class imbalance.

## 2 Related work

### 2.1 Molecular pretrained models

Pretrained language models have significantly advanced the domain of natural language processing (NLP) and addressed a myriad of challenges as exemplified by seminal works such as (Devlin *et al.* 2018). These breakthroughs inspired molecular pretrained models in the molecular science (Wang *et al.* 2019, Chithrananda *et al.* 2020). These models are adept at capturing universal molecular representations from extensive, unlabeled molecular datasets, followed by fine-tuning for specific tasks.

Initial efforts predominantly utilized sequence-based pretraining methods on molecular data represented as strings, such as SMILES (Simplified Molecular Input Line Entry System) (Tang *et al.* 2022, Liu *et al.* 2023). Prominent among these are the pioneering works of ChemBERTa by Chithrananda *et al.* (2020), SMILES-BERT by Wang *et al.* (2019), and Molformer by Ross *et al.* (2022), each contributing significantly to the understanding of molecular structures through the sequence-based representations.

Recent research has seen a marked shift towards pretraining models on molecular graphs, incorporating both 2D and 3D structures. This evolution is exemplified by studies like (Hu *et al.* 2019), which introduced techniques for masking and predicting attributes of atoms as nodes and interatom bonds as edges of molecular graphs. Liu *et al.* (2021) explored the pretraining of graph neural network (GNN) with a focus on harmonizing the alignments between 2D topological and 3D geometric features of molecules.

These developments highlight a growing interest in leveraging complex molecular graph representations for pretraining, signaling a new frontier in the integration of deep learning technique into molecular science.

### 2.2 Contrastive learning

Contrastive learning is an unsupervised learning technique that aims to learn useful representations by differentiating between similar and dissimilar samples. This approach has garnered considerable attention recently, particularly in the field of computer vision, where it has been instrumental in developing robust image representations (Park *et al.* 2020, Tian *et al.* 2020). The fundamental principle of contrastive learning involves guiding the model to generate proximal embeddings for samples within the same class, while distancing those between different classes in the embedding space (Schroff *et al.* 2015, Le-Khac *et al.* 2020).

Contrastive learning has become a technique of increasing interest to generate effective representations for the molecular property prediction task (Wang *et al.* 2022). These methods typically utilize GNNs to capture the relational information between atoms in a molecule and then employ a contrastive loss function to optimize the intraclass similarity and interclass dissimilarity in the molecular embedding space (Li *et al.* 2022, Wang *et al.* 2022).

Recent studies have ventured into multimodal molecular contrastive learning (Yang *et al.* 2021, Pinheiro *et al.* 2022). They strive to fuse information from diverse modalities to enrich the learning process. Despite its success across various research areas, contrastive learning encounters unique challenges in molecular property prediction, particularly due to limited and imbalanced data (Qi and Luo 2020).

### 2.3 Few-shot learning

Few-shot learning aims at mastering new tasks from a limited number of training samples by leveraging prior knowledge acquired during the training phase. The essence of this methodology is to cultivate models that are capable of rapidly adapting to novel tasks within minimal fine-tuning. This attribute renders them particularly advantageous in the prediction tasks with limited annotated data (Wang *et al.* 2020, Yin 2020).

Meta-learning is a popular approach of “learning to learn” and stands as a prominent strategy to facilitate few-shot learning (Liu *et al.* 2023, Lu *et al.* 2023). This technique involves training models across multiple tasks, thereby improving their ability to generalize. The model adaptability and generalization of this approach are well-documented in these works (Vilalta and Drissi 2002, Wang *et al.* 2021).

Few-shot learning has been successfully applied to a variety of molecular prediction tasks, including drug–target interactions (Altae-Tran *et al.* 2017) and molecular activities (Guo *et al.* 2021). These studies underscore the capability of few-shot learning technique to effectively counteract the challenges posted by data scarcity and class imbalance in the area of molecular science.

### 2.4 Molecular property prediction

Molecular property prediction is a critical task in many scientific areas, such as drug discovery, and material science (Shen and Nicolaou 2019). Machine learning and deep learning algorithms have recently ascended as formidable tools for predicting a spectrum of molecular properties, such as solubility, toxicity, and binding affinity (Lu *et al.* 2019, Walters and Barzilay 2020).

GNN has particularly gained prominence for its efficacy in molecular property prediction tasks through their capabilities of intricately capturing global and local information from the structure-based molecular graphs (Gilmer *et al.* 2017, Wieder *et al.* 2020). A wide range of molecular properties have been successfully predicted using various GNN architectures, such as graph convolutional network, graph attention network, and graph isomorphism network (GIN) (Duvenaud *et al.* 2015, Veličković *et al.* 2017, Xu *et al.* 2018a). Additionally, sequence-based models (Chithrananda *et al.* 2020) and 3D structure information-based models (Zhou *et al.* 2023) have also been employed for this purpose.

Despite the notable successes of these methods, limited training data and highly imbalanced class distributions persist as major challenges for molecular property prediction. Some recent studies attempted to address such challenges by integrating few-shot learning and contrastive learning techniques into molecular property predictions (Guo *et al.* 2021, Jiang *et al.* 2021, Li *et al.* 2022). However, the effective integration of these two techniques within one framework remains relatively unexplored.

## 3 Materials and methods

### 3.1 Benchmark datasets

Table 1 provides a detailed overview of the datasets employed in this study, encompassing their names, composition, and the distribution of tasks for training and testing. The benchmark datasets were sourced from the MoleculeNet database (Wu *et al.* 2018), a comprehensive and well-regarded collection of molecular datasets. These datasets were chosen due to their frequent usage in evaluating molecular property prediction models, and serve well as a robust and comparable testing environment for our study. The selection was guided by their comprehensive coverage of compound structures and diversity in prediction tasks, which are crucial for assessing the versatility of our model.

**Table 1.** Summary of datasets used in this study.

Dataset	Compounds	Tasks	Training tasks	Testing tasks
Tox21	8014	12	9	3
SIDER	1427	27	21	6
MUV	93 127	17	12	5
ToxCast	8615	617	450	167

The first column gives the names of the datasets. The columns “Compounds” and “Tasks” list the numbers of compounds and the prediction class labels of each dataset. The last two columns give the numbers of training and testing class labels, respectively.

**Table 2.** Detailed results of hyperparameter tuning.

Hyperparameter	Tested values	Impact
Learning rate	0.001, 0.0001, 0.00005	Influences convergence speed and stability
Contrastive loss weighting coefficient	0.1, 1, 10	Balances supervised and contrastive loss components
The number of training samples in the support set during the meta-training phase	3, 5, 7	Determines adaptability and efficiency

This table outlines the optimization of the hyperparameters under consideration, along with the range of values tested and their respective impacts on model performance.

**Figure 1.**
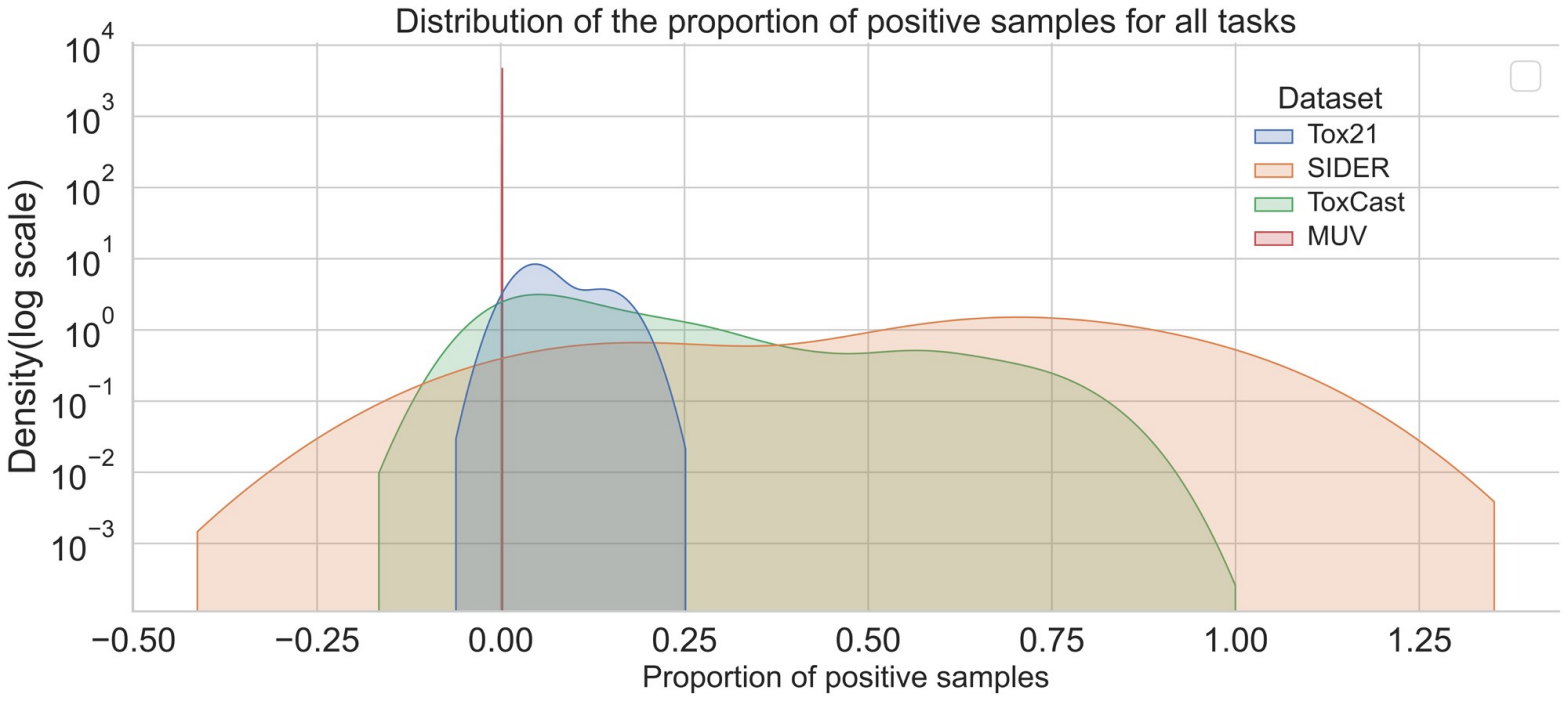
The data distribution of the proportion of positive samples for all tasks on each dataset. *X*-axis is the proportion of positive samples. *Y*-axis is the density in log scale. For this plot, we employ kernel density estimation to smooth the observations with a gaussian kernel.

Figure 1 shows that among the four datasets, the data distribution of MUV is the most imbalanced. There are only very few positive samples. Tox21 is also quite imbalanced, and the proportion of positive samples for most tasks in Tox21 is less than 20%. SIDERH and ToxCast have relatively balanced distributions, while many tasks have a proportion of positive samples higher than 50%.

We used the software RDKit (Lovrić *et al.* 2019) to convert molecular structures into molecular graphs, which were then processed by our GNN-based model. The samples of each prediction task were divided into training and testing subsets, as detailed in Table 1.

For Tox21, SIDER, and MUV, we adhered to the public task splits provided in (Altae-Tran *et al.* 2017). We applied a random partitioning method on ToxCast as described in Wang *et al.* (2021), which selected 450 tasks for meta-training and the remainder for meta-testing. This method ensures consistency and fairness in comparison with other advanced methods in the field.

Consistent with the methodology described in Wang *et al.* (2021), we assess the binary classification performance using area under the receiver operating characteristic curve (AUC) scores, which are determined based on the query set for each meta-testing task. Our experimental procedure involves conducting ten separate runs, each with a unique random seed. We then calculate the average and standard deviation of the AUC scores for all the meta-testing tasks.

### 3.2 Unsupervised pretrained models

Our framework MolFeSCue integrates large-scale pretrained models to mitigate the challenge of limited annotated data in the downstream prediction tasks. Such large-scale models were pretrained on extensive unlabeled datasets, and are designed to capture a generate representation of the molecular space. They may be subsequently fine-tuned using smaller, task-specific labeled datasets to tailor their capabilities to specific tasks.

MolFeSCue employs both sequence-based and graph-based pretrained models for their distinct and complementary strengths in capturing the latent molecular patterns. Sequence-based pretrained molecular models are similar to the transformer-based models in NLP. They effectively capture the sequential characteristics inherent in molecular data. MolFeSCue integrates the pretrained molecular model ChemBERTa (Chithrananda *et al.* 2020) to leverage the sequential patterns within the benchmark datasets. The transformer-based architecture of ChemBERTa was specifically designed to represent molecules in the SMILES-encoded strings.

Graph-based models excel in deciphering the topological structures and intrinsic properties of molecules. This study adopts a pretrained GIN model (denoted as HuGIN) based on the attribute masking strategy proposed by Hu *et al.* (2019). This approach involves random masking of nodes and edges in molecular graphs to enable the graph model to learn robust representations at the node level. Several trials were conducted to evaluate various GNN architectures, and the configuration of 5-layer GIN with a linear classification layer was identified to have the best performance for the downstream tasks.

Both pretrained models adhere to the principle of learning representations in an unsupervised manner, and are further fine-tuned by the limited labeled data in the downstream tasks. Our proposed MolFeSCue framework effectively mitigates the challenge of data scarcity in the downstream molecular property prediction tasks.

### 3.3 Loss function

The training procedure of the proposed MolFeSCue framework utilizes a bifurcated loss function comprising two elements: the supervised loss related to molecular properties, and the contrastive loss aiming at accentuating differences between positive and negative samples.

The supervised loss component is characterized as the cross-entropy loss between the predicted and the actual ground-truth labels, mathematically represented as:

(1) $$L_{\text{label}\;}=-\frac{1}{k}\sum_{i=1}^{k}\;\mathrm{CrossEntropy}\left(y_{i},{\hat{y}}_{i}\right),$$

where *k* is the number of samples, *y_i_* represents the true label, and ${\hat{y}}_{i}$ represents the predicted label.

The foundational contrastive loss is expressed as:

(2) $$L_{\text{contrastive}\;}=\sum_{i,j}y_{i,j}\cdot {D\left(i,\;j\right)}^{2}+\left(1-y_{i,\;j}\right)\cdot \max(0,\;m-{D\left(i,\;j\right)}^{2}),$$

The term *D*(*i*, *j*) is the Euclidean distance between a pair of samples *i* and *j*. The ground-truth label is denoted by *y_i_*_,_*_j_*, assigned 1 if the two samples *i* and *j* belong to the same class and 0 otherwise. The parameter *m* represents a margin that is enforced between pairs of samples from different classes to ensure that the distances between samples of different classes exceed this value.

In the context of molecular property prediction, the complexity of molecular properties and the modeling challenges like activity cliffs (Deng *et al.* 2023) often lead to the emergence of numerous hard samples (Medina‐Franco 2013). Such samples substantially complicate the task of achieving cohesive representations of the molecular feature space. In the pursuit of refining molecular property predictions, our research introduces a dynamic contrastive loss function within the MolFeSCue framework to manage the fluctuating distribution of challenging negative samples, namely hard negatives. Unlike the static nature of traditional contrastive loss functions, our dynamic approach is more attuned to the learning stages of the neural network.

We consider our loss function, $L_{\text{contra-dynamic}\;}$, as a member of a broader class of functions, $L_{f(t)\;}$, where f(t) signifies the parameters influencing the loss function’s responsiveness to training dynamics. Hence, the dynamic contrastive loss function, $L_{\text{contra-dynamic}\;}$, is formulated as:

(3) $$L_{\text{contra-dynamic}\;}=\sum_{i,j}y_{i,j}\cdot {D\left(i,\;j\right)}^{2}+\left(1-y_{i,j}\right)\cdot \max(0,\;m-{D\left(i,\;j\right)}^{2})\cdot I\left(i,\;j,\;f\left(t\right)\right),$$

where *I*(*i*, *j*) is an indicator function identifying hard negative sample pairs with a value of 1, and 0 otherwise. If we take the derivative of our dynamic contrastive loss with respect to the parameter f(t), the gradient will be:

(4) ∇ftLcontra-dynamic=∑i,j( 2yi,j·D(i, j)-2(1-yi, j)·max⁡0, m-Di, j  )·∂ Ii, j, ft. ∂ft

The inclusion of f(t) in the gradient ensures that the learning rate is adjusted based on the current training phase which highlights the adaptive nature of loss function, demonstrating how the training process is dynamically tuned by incorporating the evolving ratio of hard negatives.

Next, to determine the mathematical form of f(t), we first assume that $f(t)=\frac{\sum_{i,j\;\in \;H}e^{-D(i,\;j)}}{\sum_{i,j\;\in \;\varkappa }e^{-D(i,\;j)}}$ represents the proportion of hard negative samples to all negative samples at a specific time. Here, H presents the set of hard negative samples, and ϰ encompasses all negative samples. We speculate that during the training process, difficult samples decay exponentially, but after decaying to a certain point, they tend to stabilize. Exponential decay is widely used in many applications such as physics, engineering, economics, and biology (Bohner and Peterson 2001). Specifically, exponential decay is also frequently used in the training phase of deep learning, such as adjusting learning rates (Li and Arora 2019). Therefore, we use exponential decay as the form of our temporal function *f*(*t*),

(5) $$f\left(t\right)=\alpha_{\mathrm{start}}\times e^{-\beta t}+\alpha_{\mathrm{end}},$$

where $\alpha_{\mathrm{start}}$ and $\alpha_{\mathrm{end}}$ indicating the initial and terminal ratios of hard negatives, while β denotes a decay rate governing the transition between these ratios. This decay function is carefully crafted to progressively lessen the focus on hard negative samples, enabling a more equilibrated refinement of the model’s feature space representations.

By modulating $f\left(t\right)$, we guarantee that the gradient’s magnitude concerning hard negative samples is initially more substantial, thereby emphasizing their importance early in training. As the learning progresses, this emphasis naturally wanes, mirroring the reduction in $f\left(t\right)$ and thereby mitigating the risk of overfitting that could stem from an excessive initial focus on these hard negatives.

In summary, the overall loss function of the proposed MolFeSCue framework is defined as:

(6) $$L=L_{\text{label}}+{w\cdot L}_{\text{contra-dynamic}\;}$$

where *w* is a weighting parameter that adjusts the impact of the contrastive loss within the overall loss function.

### 3.4 Proposed contrastive augmented few-shot learning

**Figure 2.**
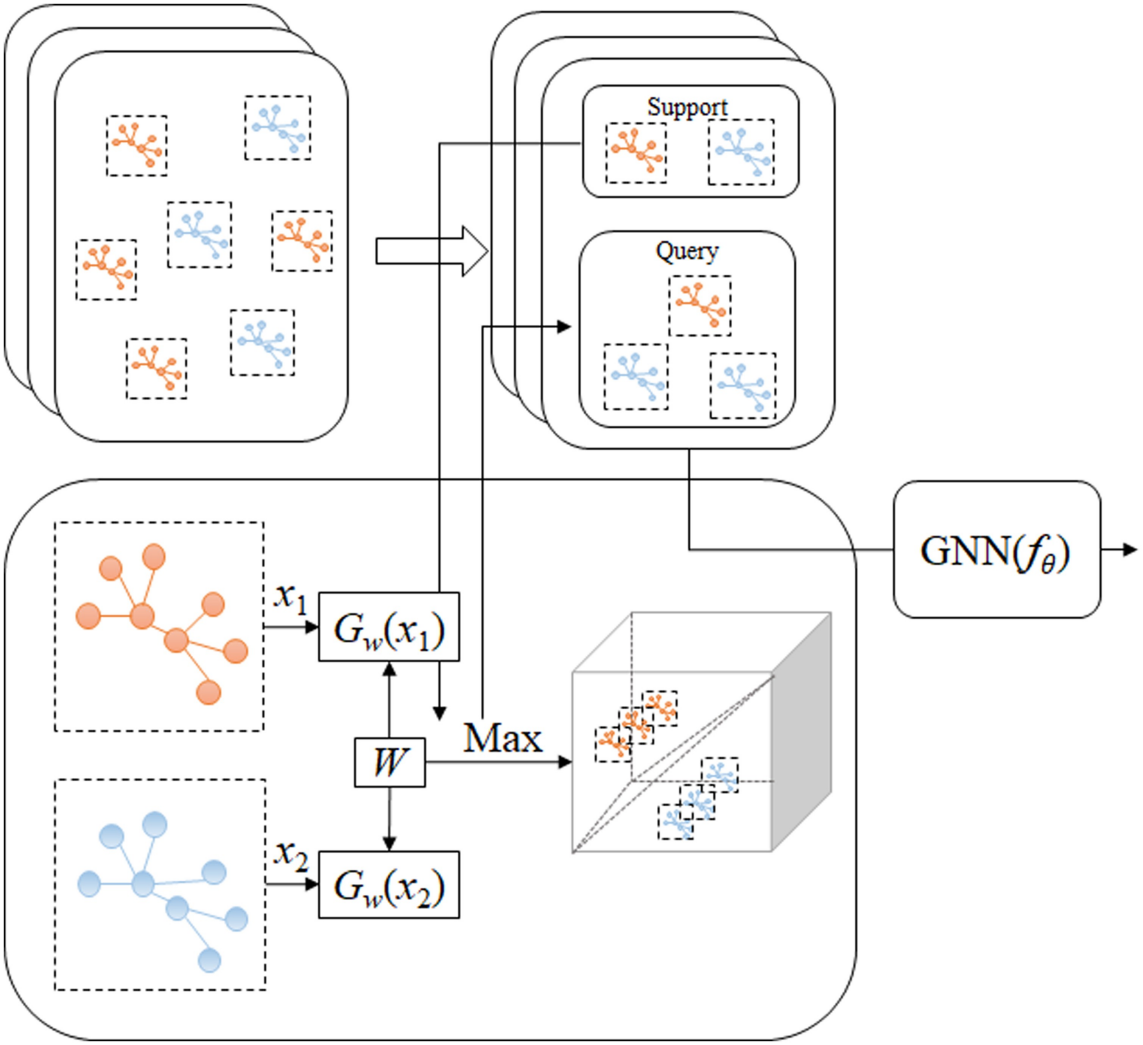
The overall architecture of the proposed framework MolFeSCue.

In addressing the prevalent challenges of data scarcity and class imbalance in molecular property prediction, the proposed framework MolFeSCue combines the strengths of few-shot contrastive learning with advanced large-scale pretrained models, as illustrated in Fig. 2. The backbone of MolFeSCue is constituted by large-scale pretrained models. This architecture provides a flexible foundation adaptable to various pretrained paradigms, including but not limited to sequence-based and graph-based models. Such a backbone also broadens the applicability of MolFeSCue across diverse molecular data types, ensuring a comprehensive approach to molecular property prediction.

Few-shot learning is the central component of MolFeSCue and is specifically engineered for rapid adaptation across various downstream tasks with a minimal dataset. MolFeSCue integrates few-shot learning with contrastive learning to enhance the differentiations between classes. Contrastive learning focuses on developing representations that cluster samples of the same class closely in the feature space while distinctly segregate the samples from different classes. This principle is particularly critical in the investigated problem of this study, where molecular properties often exhibit an activity cliff, meaning that structurally similar molecules can have very dissimilar properties. Our model amplifies this interclass distinction, effectively addressing the inherent class imbalance issues. MolFeSCue utilizes a time-dependent contrastive learning loss function *f*(*t*), which dynamically modulates the proportion parameter *α* of selected hard negatives. This loss function tunes the performance of contrastive learning, particularly in the scenario of activity cliffs, and delivers a robust and effective solution to the posed challenges.

### 3.5 Optimization of hyperparameters

We evaluated three key hyperparameters of the MolFeSCue framework, i.e. learning rate, contrastive loss weighting coefficient, and the number of few-shot meta-training samples. The selection of parameters and their impact can be seen in Table 2.

Learning rate is integral to gradient descent optimization and it dictates the pace at which the model approaches the loss function’s minimum. We experimented this hyperparameter with values 0.001, 0.0001, and 0.00005, to observe their effect on the convergence speed and stability of the model. Another hyperparameter “contrastive loss weighting coefficient” modulates the contribution of contrastive loss in MolFeSCues composite loss function. Its calibration is crucial in achieving an optimal balance between the supervised and the contrastive learning components, and thus directly impacts the model’s learning efficacy and predictive accuracy. The number of training samples in the support set during the meta training phase serves as a basis for model adaptation to new tasks, and is a critical factor in balancing sample availability with performance efficiency. We tested sample sizes of 3, 5, and 7 to determine the optimal number that ensures effective learning without compromising efficiency. It is important to recognize that different datasets may have their own optimal value choices of these hyperparameters and dataset-specific adjustments are necessary for optimal performances.

Other two hyperparameters in the MolFeSCue framework stand out in importance. First, we utilized the Jumping Knowledge Network (JK) (Xu *et al.* 2018b) to facilitate the good performance of the GIN in extracting the graph representations. We evaluated four pooling methods for the jumping layer, i.e. last, sum, max, and concat. The experimental data showed that the sum pooling was chosen for its effectiveness in aggregating the semantics of each node-level embedding for generating comprehensive graph-level embeddings. Second, the hyperparameters alpha_start, alpha_end, and beta also play critical roles. Alpha_start and alpha_end define the initial and final ratios of hard negatives, respectively, while beta represents a decay rate that controls the transition between these ratios. This decay function is carefully designed to gradually reduce the emphasis on hard negative samples, and facilitates a more balanced refinement of the model’s feature space representations. The values for these three hyperparameters are set to 1, 0.2, and 0.01, respectively.

## 4 Results and discussion

### 4.1 Performance evaluation on benchmark datasets

This section showcases the performance of our proposed MolFeSCue in comparison to the existing methods across four benchmark datasets, i.e. Tox21, SIDER, MUV, and ToxCast. Table 3 shows the results of 10-shot and 1-shot learning experiments.

**Table 3.** Performance of our proposed framework MolFeSCue and the baseline methods on the benchmark datasets.

Method	Tox21		SIDER		MUV		ToxCast	
	10—shot	1—shot	10—shot	1—shot	10—shot	1—shot	10—shot	1—shot
Siamese	80.40 (0.35)	65.00 (1.58)	71.10 (4.32)	51.43 (3.31)	59.96 (5.13)	50.00 (0.17)	–	–
ProtoNet	74.98 (0.32)	65.58 (1.72)	64.54 (0.89)	57.50 (2.34)	65.88 (4.11)	58.31 (3.18)	63.70 (1.26)	56.36 (1.54)
MAML	80.21 (0.24)	75.74 (0.48)	70.43 (0.76)	67.81 (1.12)	63.90 (2.28)	60.51 (3.12)	66.79 (0.85)	65.97 (5.04)
TPN	76.05 (0.24)	60.16 (1.18)	67.84 (0.95)	62.90 (1.38)	65.22 (5.82)	50.00 (0.51)	62.74 (1.45)	50.01 (0.05)
EGNN	81.21 (0.16)	79.44 (0.22)	72.87 (0.73)	70.79 (0.95)	65.20 (2.08)	62.18 (1.76)	63.65 (1.57)	61.02 (1.94)
IterRefLSTM	81.10 (0.17)	80.97 (0.10)	69.63 (0.31)	71.73 (0.14)	49.56 (5.12)	48.54 (3.12)	–	–
PAR	82.06 (0.12)	80.46 (0.13)	74.68 (0.31)	71.87 (0.48)	66.48 (2.12)	64.12 (1.18)	69.72 (1.63)	67.28 (2.90)
Pre—GNN	82.14 (0.08)	81.68 (0.09)	73.96 (0.08)	73.24 (0.12)	67.14 (1.58)	64.51 (1.45)	73.68 (0.74)	72.90 (0.84)
Meta—MGNN	82.97 (0.10)	82.13 (0.13)	75.43 (0.21)	73.36 (0.32)	68.99 (1.84)	65.54 (2.13)	–	–
Pre—PAR	84.93 (0.11)	**83.01 (0.09)**	78.08 (0.16)	**74.46 (0.29)**	69.96 (1.37)	66.94 (1.12)	75.12 (0.84)	73.63 (1.00)
Ours	**85.93 (0.10)**	82.05 (0.11)	**79.08 (0.14)**	73.13 (0.56)	**72.96 (1.18)**	**67.32 (1.08)**	**76.39 (1.52)**	**74.82 (1.39)**

The performance metric is AUC. The mean and standard deviation of the AUC values over 10 random runs are given in each grid and in parenthesis. The best results are highlighted in bold.

Ten baseline methods are chosen for the comparative experiment. We conduct a comparison between our MolFeSCue system and two groups of baseline methods. The first group includes few-shot learning (FSL) methods that utilize a graph-based molecular encoder developed from scratch. This group comprises various approaches such as Siamese (Koch *et al.* 2015), ProtoNet (Snell *et al.* 2017), MAML (Finn *et al.* 2017), TPN (Wang et al. 2022), EGNN (Kim *et al.* 2019), and IterRefLSTM (Altae-Tran *et al.* 2017), along with PAR (Wang *et al.* 2021). The second group consists of methods that employ a pretrained graph-based molecular encoder. These methods include Pre-GNN (Hu *et al.* 2019), Meta-MGNN (Guo *et al.* 2021), and Pre-PAR (Wang *et al.* 2021), which is a combination of PAR with Pre-GNN. For the experimental results of baseline models, we directly cite the outcomes presented in Wang *et al.* (2021).

Our method outperforms the other algorithms in most of the cases in Table 3. MolFeSCue achieves the best AUC values on both 10-shot and 1-shot learning of the two datasets MUV and ToxCast. An impressive AUC of 85.93% in the 10-shot scenario is achieved by MolFeSCue on the dataset Tox21. Overall, MolFeSCue performs the best on two datasets MUV and ToxCase in the 1-shot scenario, and is ranked as the best on all the four datasets in the 10-shot scenario.

The other methods show notable variations across different datasets and scenarios. The Pre-PAR method outperforms MolFeSCue on the two datasets Tox21 and SIDER under 1-shot condition. However, Pre-PAR only achieves 69.96% and 66.94% in AUC under the 10-shot and 1-shot conditions. So MolFeSCue achieves the best mean AUC of 76.46% and the best mean rank 1.63.

MolFeSCue exhibits a significant advantage in computational efficiency through its few-shot learning approach, which notably minimizes the computational burden while ensuring high accuracy. This is highlighted by the contrast with non-few-shot frameworks like Siamese and EGNN, which generally require larger sample sets for model training. The meta-testing phase of MolFeSCue, for example, requires only a small number of samples for training, thus reducing training time and computational resources.

Another crucial aspect of MolFeSCue is its scalability, demonstrated through its consistent performance across datasets with varying sizes and complexities. For example, on the ToxCast dataset, which contains a large number of compounds and prediction tasks, MolFeSCue maintains an average AUC score of 76.39%, outperforming other methods such as Pre-PAR and EGNN (Table 3). This indicates its capability to handle large-scale data efficiently, and makes it highly applicable in diverse real-world scenarios such as drug discovery and toxicity prediction, where dataset size and complexity are common challenges. Incorporating the performance on the SIDER dataset, we observe that while our AUC in one-shot conditions slightly trails Pre-PAR, MolFeSCue excels in 10-shot scenarios, achieving the highest AUC. This reflects a crucial aspect of drug development, where data typically accumulates over time. Ideally, a model’s performance improves as more data become available, a trend that MolFeSCue exemplifies. The experimental data demonstrate their suitability for evolving and data-rich environments in pharmaceutical research.

Figure 1 shows that MUV is an extremely imbalanced dataset, with as many as 93 127 samples. On this dataset, MolFeSCue achieved the greatest improvement, reaching 4%. Moreover, on the MUV dataset, MolFeSCue is the only model that surpasses a AUC of 0.7. The performance of MolFeSCue on such extremely imbalanced datasets demonstrates its ability not only to recall positive samples but also to eliminate false positives from a large molecular library. This indicates the potential of MolFeSCue for practical application in virtual screening.

In scenarios with more balanced sample sizes, such as the SIDER or ToxCast datasets, MolFeSCue also performs better than other baseline models. This shows that MolFeSCue is not only adept at handling imbalanced datasets but also performs well on balanced datasets. Interestingly, although Tox21 is imbalanced, MolFeSCue achieved AUC scores of 82.05 and 85.93 under 1-shot and 10-shot conditions, respectively, which is even better than the performance on the other two relatively balanced datasets. Therefore, the experimental results suggest that MolFeSCue is sufficiently robust, with minimal impact from the distribution of positive and negative samples in the training data.

### 4.2 Effectiveness of pretrained models

**Figure 3.**
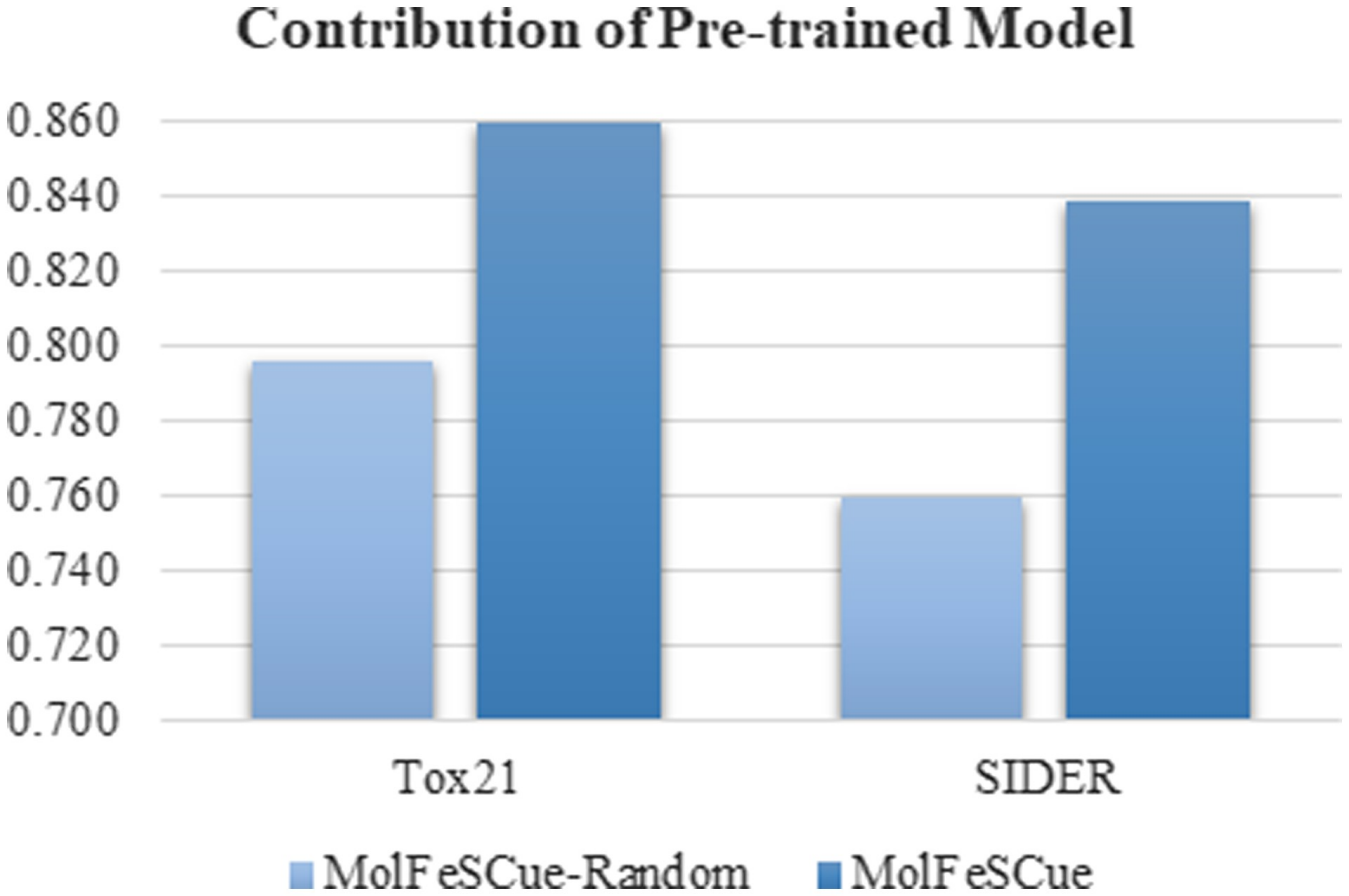
AUC scores achieved by MolFeSCue and MolFeSCue-Random on the Tox21 and SIDEr datasets. MolFeSCue represents the full version of our proposed model, while MolFeSCue-Random denotes the variant of MolFeSCue without the pretrained parameters for initializing the graph neural network. All the results are based on the 10-shot scenario.

We conduct an ablation experiment to ascertain the impact of the pretrained models on addressing the challenge of limited annotated data in the downstream molecular property prediction tasks, as shown in Fig. 3. The full version of MolFeSCue integrates large-scale pretrained models, while the ablated counterpart MolFeSCue-Random does not utilize the pretrained models.

The full version of MolFeSCue attains notable AUC scores of 0.859 on the Tox21 dataset and 0.838 on the SIDER dataset. The performance of the ablated versions MolFeSCue-Random is drastically decreased to the AUC scores of 0.796 and 0.759 on the Tox21 and SIDER datasets, respectively.

Figure 3 underscores the pivotal role of the pretrained models in enhancing the prediction performance of MolFeSCue. The substantial performance boost provided by these pretrained models demonstrates their efficacy in enabling more precise molecular property prediction.

### 4.3 Effectiveness of contrastive augmented few-shot learning

**Figure 4.**
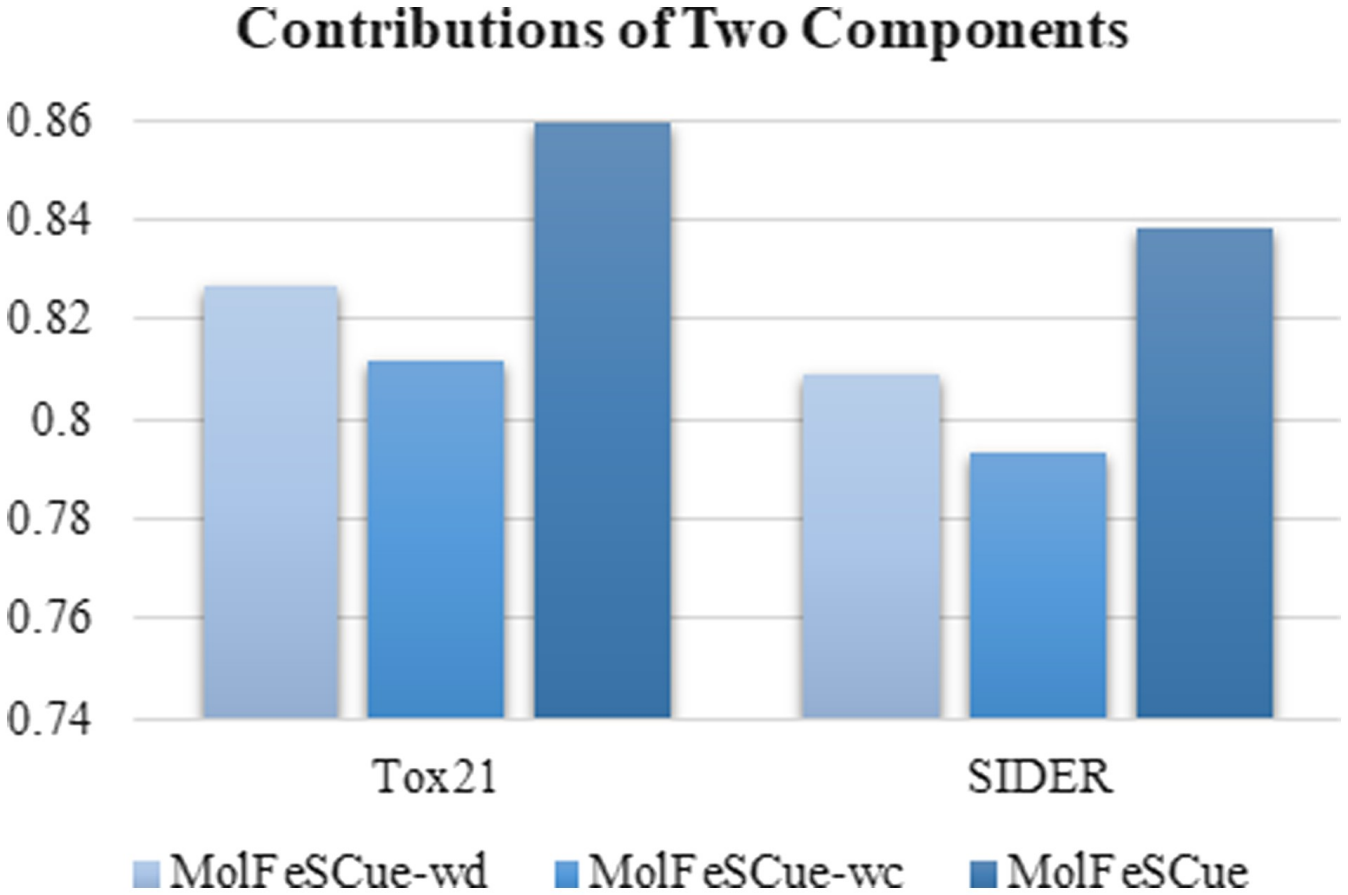
AUC scores for the three architectures. MolFeSCue represents the full version of our proposed method. MolFeSCue-wc excludes the contrastive loss from the loss function of MolFeSCue. MolFeSCue-wd does not utilize the dynamic adjustment of the negative sample rate *f*(*t*) in its contrastive learning component. All the results are obtained under the 10-shot learning condition.

This section evaluates the contributions of two components to the proposed MolFeSCue framework. One variant MolFeSCue-wc removes the contrastive loss from the loss function of MolFeSCue. The other variant MolFeSCue-wd does not include the dynamic adjustment of the negative sample rate. The detailed comparison is illustrated in Fig. 4.

The full version of MolFeSCue outperforms the two variants MolFeSCue-wc and MolFeSCue-wd on the two datasets Tox21 and SIDER. The best AUC score 0.859 is achieved by MolFeSCue on the Tox21 dataset, while the best AUC score is 0.838 on the SIDER dataset.

The experimental data in Fig. 4 lead us to two conclusions. First, the significance of contrastive learning in the molecular property prediction task is unmistakably evident (Pinheiro *et al.* 2022, Wang *et al.* 2022, Liu *et al.* 2023). Its effectiveness in addressing challenges related to data scarcity and class imbalance is highlighted by the substantial drop in performance observed in the MolFeSCue-wc model, which lacks the contrastive loss. Second, the performance gap between MolFeSCue and MolFeSCue-wd accentuates the importance of dynamically adjusting the negative sample rate in the MolFeSCue framework.

In summary, the experimental data provide compelling evidence that both contrastive learning and dynamic sample rate adjustment play essential roles in enhancing the effectiveness of MolFeSCue for molecular property prediction.

### 4.4 Further exploration of few-shot learning

**Figure 5.**
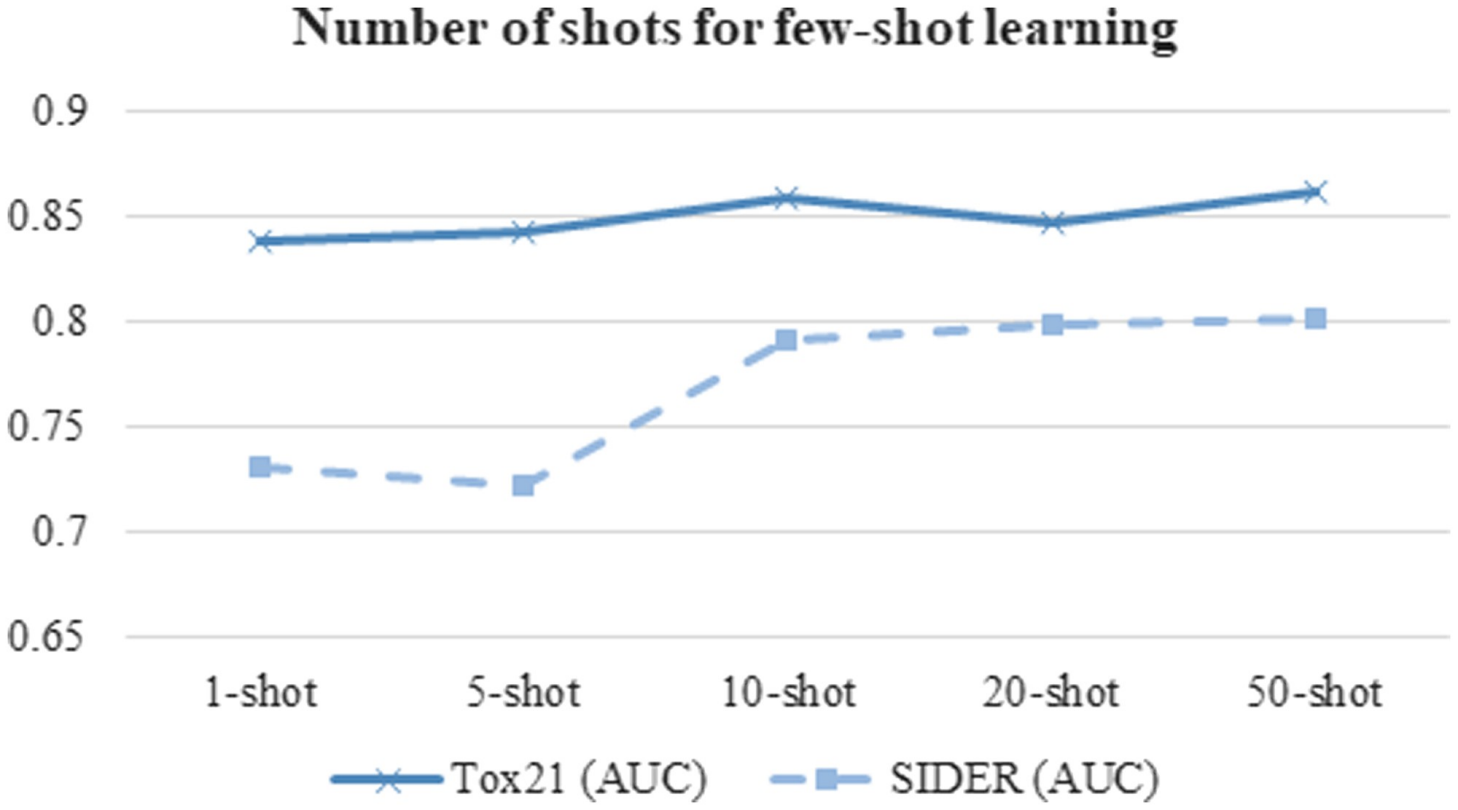
Performance evaluation of the proposed MolFeSCue framework with different numbers of shots (k) for the few-shot learning. The horizontal axis lists the evaluated numbers of shots for the training process of few-shot learning. The vertical axis gives the overall prediction AUC scores of the evaluated models. The performance is calculated on the datasets Tox21 (solid line) and SIDER (dashed line).

We further investigate how the number of shots influences the efficacy of the MolFeSCue framework in molecular property prediction (Fig. 5). The term “shots” here refers to the number of labeled samples in each class for the training process of the downstream tasks. A series of experiments on the Tox21 and SIDER datasets are conducted to evaluate the different numbers of shots for the prediction tasks. A particular emphasis is laced on the challenging scenario where there is only one shot, meaning only one labeled sample per class is available for training a prediction model of the downstream task. The experiment aims to evaluate the robustness of our MolFeSCue framework in extremely data-scarce conditions.

Figure 5 illustrates that there is a discernible trend where an increase in the number of shots correlates with an improvement in the MolFeSCues performance. Even in scenario of one-shot learning, MolFeSCue manages to deliver promising results. Specifically, MolFeSCue achieves AUC scores 0.838 and 0.731 on the Tox21 and SIDER datasets even for the one-shot learning setting. This underscores its proficiency in utilizing minimal labeled data effectively. The data suggest that MolFeSCue can make accurate predictions even in the most data-constrained scenarios, and its performance is further enhanced as the number of available labeled samples increases.

### 4.5 Generalized to sequence-based pretrained model

The experimental results illustrate that our MolFeSCue framework extends across sequence-based and graph-based molecular representation pretrained models. This adaptability underscores the MolFeSCue framework’s versatility and robustness, evidenced by its sustained efficacy across different model architectures.

Table 4 details the performance of MolFeSCue and its variant MolFeSCue-seq. A slight reduction in AUC scores was observed when transitioning from the full version MolFeSCue to the sequence-based variant (MolFeSCue-seq). Notably, MolFeSCue-seq achieves a remarkable ROC-AUC score of 0.734 on the MUV dataset, which is much better than the second-best algorithm in Table 3.

**Table 4.** AUC scores between MolFeSCue and MolFeSCue-seq.

Method	Tox21		SIDER		MUV		ToxCast	
	10—shot	1—shot	10—shot	1—shot	10—shot	1—shot	10—shot	1—shot
MolFeSCue	**85.93**	**82.05**	**79.08**	**73.13**	72.96	**67.32**	**76.39**	**74.82**
MolFeSCue-seq	84.19	79.68	78.2	70.21	**73.41**	55.74	74.28	70.55

MolFeSCue is the full version of our proposed model, while MolFeSCue-seq only employs the sequence-based pretrained model. The performance is evaluated using the AUC score. The best value in each column has been highlighted in bold.

In summary, the MolFeSCue framework can be easily generalized to integrate the other pretrained models. Table 4 shows that the sequence-based pretrained model achieves reasonable performance of the molecular property prediction task.

### 4.6 Feature embedding analysis and interpretability

To analyze the representations learned by MolFeSCue, we utilized principal component analysis (PCA) (Wold *et al.* 1987) to reduce the dimensions of the features learned by MolFeSCue to a 2D space, which we then visualized. Additionally, we conducted a comparison with a pretrained GIN model trained using the same few-shot training process but without the contrastive augmented few-shot learning framework.

**Figure 6.**
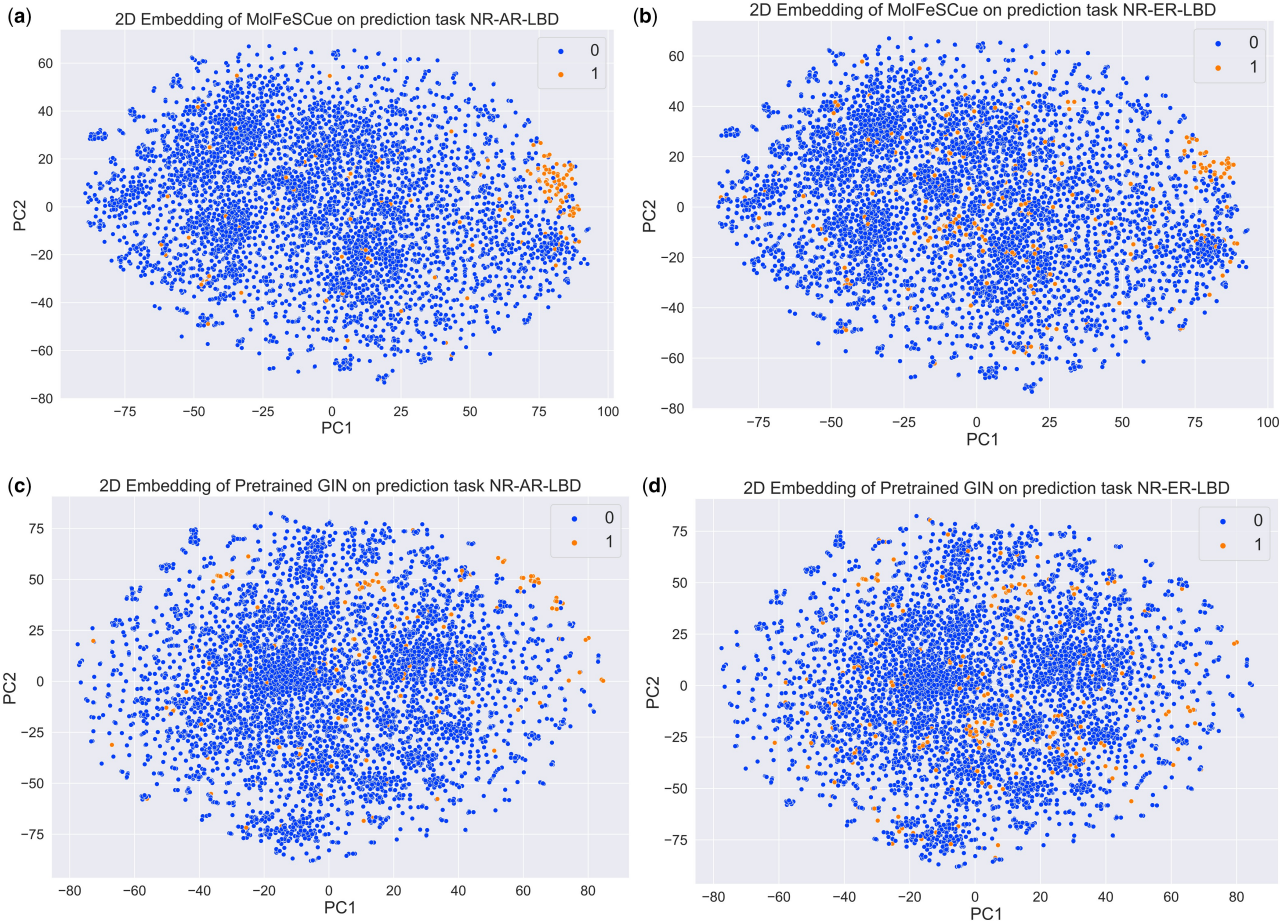
Comparison of MolFeSCue and pretrained GIN on two tasks, NR-AR-LBD and NR-ER-LDB, in the Tox21 dataset. *X*-axis is principle component 1. *Y*-axis is principle component 2 (a) Representations learned by MolFeSCue on the NR-AR-LBD task. (b) Representations learned by MolFeSCue on the NR-ER-LBD task. (c) Representations learned by pretrained GIN on the NR-AR-LBD task. (d) Representations learned by pretrained GIN on the NR-ER-LBD task.

From the observations depicted in Fig. 6a and b, a distinct separation in the distribution of data points for class 1 and class 0 in the 2D PCA space of the MolFeSCue embeddings is shown. Despite the limited number of molecules in class one, their representations are concentrated in the upper-right corner. Conversely, in Fig. 6c and d, molecules from both classes exhibit a higher degree of overlap in the 2D PCA space of the pretrained GIN embeddings, making it challenging to define a clear decision boundary.

These findings indicate that the representations learned by MolFeSCue possess enhanced discriminative capabilities across different classes. Remarkably, the model has the ability to discern inherent differences among these molecules, even when it has not been explicitly trained on labels associated with these samples. This adaptability enables MolFeSCue to rapidly apply its acquired knowledge to new prediction tasks, resulting in superior classification performance.

## 5 Conclusion and future directions

This study introduced a novel few-shot contrastive learning framework MolFeSCue for molecular property prediction in data-scarce and imbalanced scenarios. MolFeSCue synergistically combines few-shot learning strategy with contrastive learning loss to address the dual challenges of limited data availability and class imbalance. The efficacy of our framework is rigorously validated on benchmark datasets, where MolFeSCue consistently outperforms several baseline models. Ablation experiments further elucidate the crucial roles of pretrained models, contrastive augmented few-shot learning, and dynamic adjustment of hard negative sample rates in enhancing MolFeSCues prediction power. The exploration of the number of training shots underscores the framework’s robustness in delivering remarkable results even with minimal labeled data.

However, the combination of few-shot and contrastive learning in MolFeSCue increases model complexity, which leads to longer training times and elevated computational requirements. This complexity may limit its deployment in resource-constrained settings. Additionally, while our framework excels in toxicity-related property predictions (Tox21, SIDER, ToxCast), its effectiveness across a broader range of molecular properties remains to be explored. Specifically, properties like binding affinity, which rely not just on molecular structure but also on protein interactions, pose a challenge for MolFeSCue, given its reliance solely on molecular data. The discontinuous nature of the property space and the presence of activity cliffs further complicate accurate property prediction with limited molecular inputs, highlighting the need for further validation of our model’s applicability in real-world drug discovery processes.

Addressing these limitations, it is crucial to consider the diversity of pretrained models. While MolFeSCues validation includes two types of pretrained models, others like SELFormer offer potential for future explorations (Yüksel *et al.* 2023). SELFormer is a breakthrough chemical language model using SELFIES notation for flexible and high-quality molecular representation. Its success underscores the need for further research on MolFeSCues compatibility with a variety of pretrained models, including those from different paradigms. Moreover, the integration of molecular dynamics simulations and machine learning, as evidenced by recent advances in computational tools for drug design, suggests an alternative avenue for improving model accuracy in specific scenarios (Xia *et al.* 2023). These methods combine physical principles with machine learning, and may provide superior outcomes by enhancing small molecule representation learning and efficient exploration of chemical space. Therefore, in the actual prediction of molecular properties or the scenario of new drug development, combining MolFeSCues powerful representation learning ability with the first-principles molecular dynamics of physics may produce better results.

In summary, our MolFeSCue framework represents a novel advancement in molecular property prediction, particularly in challenging scenarios characterized by data scarcity and class imbalance. Future research could focus on extending the application of our framework to a wider array of molecular property prediction tasks. Investigating additional computational techniques to further enhance performance in data-limited contexts remains an exciting avenue. We are optimistic about MolFeSCues potential to be adapted for various applications in drug discovery, toxicity assessment, and the wider pharmaceutical and chemical industries, although its practical utility and generalizability require further validation in the face of discontinuous property spaces and activity cliffs.

## Supplementary Material

btae118_Supplementary_Data
